# Bidirectional Association Between Internet Use and Depressive Symptoms Among Middle-Aged and Older Adults in China: A Cross-Lagged Model of Proactive Health Behavior as the Mediating Role

**DOI:** 10.1155/da/9391682

**Published:** 2025-09-22

**Authors:** Zhibin Li, Huijun Liu

**Affiliations:** ^1^School of Public Policy and Administration, Xi'an Jiaotong University, Xi'an, China; ^2^Center for Ageing and Health Research, School of Public Policy and Administration, Xi'an Jiaotong University, Xi'an, China

**Keywords:** bidirectional association, cross-lagged model, depressive symptoms, internet use, proactive health behavior

## Abstract

**Objectives:** The present study aimed to examine the bidirectional relationship between internet use and depressive symptoms among middle-aged and older adults. Moreover, it explored whether proactive health behavior mediates the association between internet use and depressive symptoms.

**Methods:** We used the latest three-wave data (2015, 2018, and 2020) from the China Health and Retirement Longitudinal Study (CHARLS), which included 11,332 participants aged 45 years and older. The bidirectional relationship between internet use and depressive symptoms was examined using a cross-lagged model. The mediating role of proactive health behavior was also investigated using a cross-lagged mediation model.

**Results:** Cross-lagged models indicated reciprocal effects between depressive symptoms and internet use. Internet use had a greater impact on subsequent depressive symptoms than vice versa. Mediation analyses further revealed that proactive health behavior significantly mediated the path from internet use to depressive symptoms. Furthermore, subgroup analyses showed these effects were not significantly heterogeneous in subgroups by age and chronic disease status.

**Conclusions:** This study sheds light on the direction of the association between internet use and depressive symptoms. Internet use could reduce depressive symptoms among middle-aged and older adults by enhancing proactive health behavior.

## 1. Introduction

Depression in later life not only severely influences the quality of life of sufferers, but can also lead to cognitive decline, social isolation, increased utilization of medical resources, and even heightened risk of suicide [1]. A systematic review and meta-analysis disclosed that the global prevalence of depression in older adults was 28.4% [2]. In China, the prevalence of depressive symptoms among middle-aged and older adults is also equally concerning; it has remained relatively high, ranging from 32% to 37% between 2008 and 2015 [3, 4]. Therefore, examining the influencing factors of depressive symptoms could significantly inform the development of policies and interventions aimed at mitigating the risk of depression among middle-aged and older adults in China.

In the digital age, the rapid development of internet technology and its application in promoting healthy aging provide new opportunities for improving the mental health of middle-aged and older adults [5]. The internet is a versatile tool, offering various functions for users, including access to health-related information, online shopping, video entertainment, and maintaining close contact with distant relatives and friends [6–8]. These diverse functionalities change the lifestyle of middle-aged and older adults and help them overcome temporal and spatial barriers, enhancing their connectivity with the outside world [9]. In the meantime, internet use expands middle-aged and older adults' social networks [10], boosts social capital [11], reduces the likelihood of suffering from social isolation [12], and contributes to health and well-being [8, 13].

Previous studies have shown inconsistent associations between internet use and depressive symptoms. A study in China reported that older adults who used internet had lower depression levels than older adults who did not use it [14]. A US study also observed this similar correlation among retired adults aged 50 years and older [9]. However, some studies suggest that internet use has an adverse impact on depression, increasing depressive tendencies in older adults [15]. Additionally, a study indicates a lack of significant correlation between internet use and depressive symptoms [16]. Researchers commonly attribute the inconsistency in research findings to issues with sample selection, regional disparities, and methodological differences [17, 18].

Nevertheless, these studies ignored the possible bidirectional relationship between internet use and depressive symptoms. A recent survey of older adults found that depression hinders older adults from internet adoption [19]. Liu et al. [20] reported that longer internet use time predicted lower levels of depressive symptoms; similarly, higher levels of depressive symptoms significantly predicted less internet use time. In contrast, Cotten et al. [21] found that social media use and depressive symptoms do not influence each other. Differences in data and national settings may account for discrepancies in findings across studies. Besides, scholars have posited that two-wave data within longitudinal research paradigms is inadequate for achieving methodological rigor and potentially undermines the credibility of the findings [22]. Therefore, there is an urgent need for further longitudinal research to clarify whether a bidirectional relationship exists among middle-aged and older adults in Chinese settings.

Moreover, the underlying mechanisms between internet use and depressive symptoms among middle-aged and older adults need to be explored in depth. Based on the social connections' function of internet, previous research has revealed various pathways, such as structural social capital [23], social isolation [24], and formal social participation [25]. Beyond the function of social connections, as a convenience, immediacy, and diversity of information sources, the internet is increasingly becoming a crucial channel through which individuals can obtain health-related information and knowledge [26]. Research indicates that a primary motivation for older adults to use internet is to seek health-related information [27]. Health-related knowledge and information can help older adults adopt a healthy lifestyle and engage in proactive health behaviors [28]. Taking the function of the information source of the internet into consideration, proactive health behavior may be a potentially significant mediator that has not been extensively studied.

In China, the strategic concept of “proactive health” has led to more discussion on the relationship between proactive health behavior and health. As a novel health paradigm, proactive health emphasizes the need to shift the focus of health initiatives upstream, value individuals' independence and agency, and prioritize their active and ongoing behavioral engagement [29]. Researchers have incorporated proactivity into the health research framework, highlighting the importance of active prevention and intervention in health promotion [30–34]. Studies revealed that proactive health behavior was a crucial protective factor for the health and well-being of older adults. For example, leisure-time physical activity and fruit or vegetable consumption predict subsequent lower depressive symptoms among older Taiwanese individuals [35]. Older adults who consistently engage in more healthy behaviors have been found to experience higher levels of well-being and be at lower risk of developing mental health issues [36].

Nevertheless, empirical research on the relationship between internet use and proactive health behavior is limited, and the conclusions are inconsistent. For example, a study in the United Kingdom indicated that older adults who used internet were more likely to undergo preventive colorectal cancer screening [37]. Compared to noninternet users, middle-aged and older adults who frequently used internet were more likely to adopt an overall healthy lifestyle [38]. In contrast, some studies emphasize that internet use does not necessarily lead to proactive health behavior and healthy lifestyles. A study on Polish older adults found no association between internet use and a healthy lifestyle [39]. A systematic review and meta-analysis found that internet-based interventions had limited and statistically insignificant effects on changing health behavior [40].

Taken together, the existing research on the relationship between internet use and depressive symptoms is characterized by several limitations: First, prior studies have predominantly focused on older adults, paying limited attention to middle-aged cohorts who are typically more active internet users yet may also be at risk of early mental health decline. In addition, important health-related covariates such as chronic disease status, which is strongly associated with depressive symptoms, have often been overlooked in subgroup analyses. Second, most existing studies adopted cross-sectional designs, limiting the ability to assess the causal and bidirectional relationships between internet use and depressive symptoms. Limited longitudinal studies have mainly been conducted in developed countries. Finally, the potential mediating effect of proactive health behavior on the relationship between internet use and depressive symptoms has not been extensively explored, particularly in longitudinal research.

Therefore, we used the latest three waves (2015–2020) data from the China Health and Retirement Longitudinal Study (CHARLS) and employed the cross-lagged panel model to investigate the longitudinal and bidirectional relationships between internet use and depressive symptoms among middle-aged and older adults. Furthermore, we explored the mediating effect of proactive health behavior on this relationship, shedding new light on the dynamic interplay between internet use, depressive symptoms, and proactive health behavior. Additionally, we conducted subgroup analyses by age and chronic disease status to better capture heterogeneity in these associations.

## 2. Methods

### 2.1. Sample and Produce

We used data from the CHARLS conducted by Peking University and approved by the Biomedical Ethics Committee of Peking University (Review Grant Number: IRB00001052-11015), and the study data were anonymous. Each participant provided signed informed consent at the time of participation. The CHARLS is a nationally representative longitudinal survey of community-dwelling persons aged 45 years and older in China, involving assessments of their social, economic, and health status. A more thorough description of the CHARLS study design and sample techniques has already been published [41]. More information could be found in this official website (https://charls.pku.edu.cn/). Given the insufficient number of internet users in Wave 1 (2011) and Wave 2 (2013) and poor timeliness, we used panel data from the latest waves, including Wave 3 (T1, 2015), Wave 4 (T2, 2018), and Wave 5 (T3, 2020).

**Figure 1:**
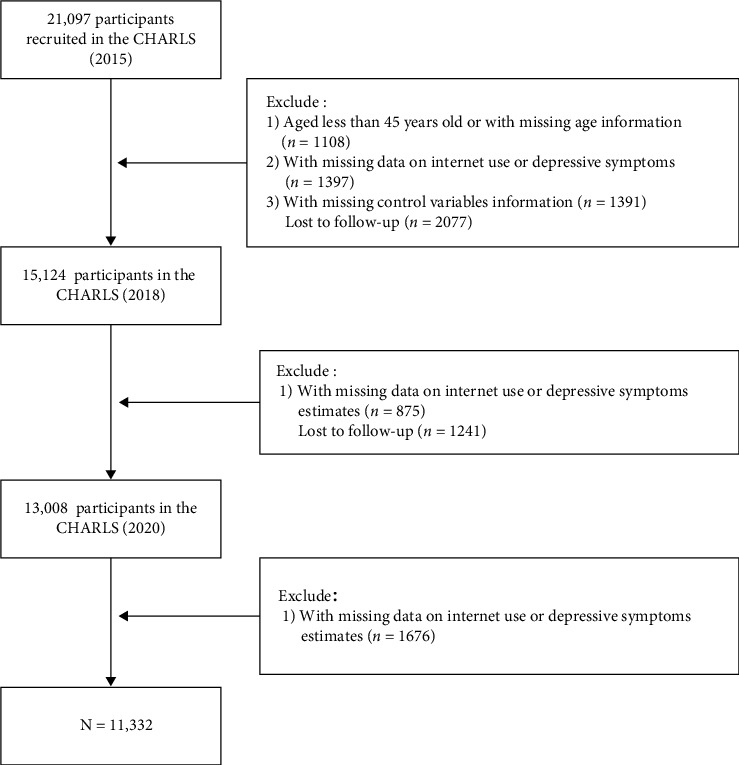
Sample selection criteria and procedure in this study.

We restricted the analysis sample to persons aged at least 45 years in 2015, who were followed up in the two subsequent waves. We further screened the sample based on the criteria presented in Figure 1. Participants who completed three follow-up surveys and had no missing values on depressive symptoms, internet use, and control variables (T1) were retained. Ultimately, 11,332 middle-aged and older adults were included in our study.

### 2.2. Measures

#### 2.2.1. Depressive Symptoms

Depressive symptoms were measured by the 10-item Center for Epidemiologic Studies Depression scale (CES-D-10) [42], including eight negative items and two positive items. The respondents were asked how often they had felt and behaved (e.g., loneliness, happiness, feeling doing everything is very difficult, and so on) during the last week. Responses were coded on a 4-point Likert-type scale (0 = never; 3 = most of the time). The scores of two positive items were inverted to keep the same direction with the total scale. Finally, the scores were summed, ranging from 0 to 30, with higher scores indicating a higher level of depressive symptoms. The Cronbach's *α* values of the measure of depressive symptoms were 0.800, 0.840, and 0.846 for T1, T2, and T3, respectively. To ensure the psychometric soundness of this approach within our sample, we conducted a confirmatory factor analysis (CFA) to test the structural validity of the scale for each wave of data. The results showed a good model fit across all three waves (Table S1).

#### 2.2.2. Internet Use

In the CHARLS survey, respondents were asked, “Have you used internet in the past month?” Following previous studies [9, 13] if the answer was “yes,” they were classified as internet users and assigned a value of 1. Otherwise, they were assigned a value of 0. To assess the robustness of our findings, we introduced digital exclusion as an alternative exposure variable in the sensitivity analysis. Digital exclusion was defined based on two questions: (1) “Have you used the internet in the past month?” and (2) “Does this household have internet access?” Respondents answering “no” to either question were classified as “digitally excluded” (coded as 0), otherwise as “nondigitally excluded” (coded as 1).

#### 2.2.3. Proactive Health Behavior

Building on the analysis and understanding of the connotation of proactive health from existing research [29, 31, 43], this study conceptualized proactive health behavior as a formative construct, encompassing two dimensions: healthy lifestyles and self-health management.

Healthy lifestyles mainly include sleep duration, smoking consumption, and alcohol consumption. The measure of sleep duration was based on the following question: “During the past month, how many hours of actual sleep did you get at night (average hours for one night)?” According to the sleep duration recommendations of the National Sleep Foundation [44], the reported sleep duration was divided into three categories: recommended (7–9 h), appropriate (9–10 h and 6–7 h), and not recommended (less than 6 h and more than 10 h). Three categories were assigned of values “3,” “2,” and “1,” respectively. The measure of smoking consumption was based on the question: “In one day, how many cigarets do you consume now?” Smoking, regardless of its amount, is detrimental to health [45]. However, considering the potential subtle variations in smoking habits, following previous studies [46], we recorded the “number of cigarets smoked daily” as an ordinal variable from 1 to 4, representing the level of smoking (4 = nonsmoker at present; 3 = smoked 1–10 cigarets per day; 2 = smoked 11–20 cigarets per day; 1 = smoked more than 20 cigarets per day).

The measure of alcohol consumption was based on the following questions. First, in the 2015, 2018, and 2020 surveys, participants were asked the same question: “Did you drink any alcoholic beverages, such as liquor, beer, or wine, in the past year?” If the participant did not consume alcoholic beverages in the past year, they were identified as “nondrinkers.” Those who indicated that they drank any one of three types of alcohol (liquor, beer, or wine) in the last year were identified as “current drinkers.” Next, all current drinkers were further divided based on their reported alcohol consumption frequency. In the 2015 and 2018 surveys, they were further asked, “How often did you drink three types of alcoholic beverages (beer, wine, or liquor) per month last year?” However, in the 2020 survey, the three questions were integrated into one question, “How often did you drink any one of three types of alcohol (liquor, beer, or wine) per month in the past year?” We followed the methods of Harmonized CHARLS [47]; for the 2015 and 2018 surveys, we utilized the highest range of drinks per day that the respondent reported for any of the three types of alcohol (liquor, beer, or wine). For the 2020 survey, we directly adopted the reported alcohol consumption frequency of participants. Finally, following previous studies [48], we recorded alcohol consumption into an ordinal variable from 1 to 4 representing the level of drinking (4 = nondrinker; 3 = drank less than once per day; 2 = drank once per day; 1 = drank at least twice per day).

Self-health management is a key component of proactive health behavior, encompassing actions individuals take to maintain or improve their health, specifically through participation in physical examinations and regular physical exercise [49, 50]. The measurement of self-health management is based on two binary indicators: participation in physical examination and engagement in regular physical exercise. These two binary indicators contribute to a composite proactive health behavior index by reflecting individual engagement in preventive health practices. Specifically, the measure of physical examination, as detailed below: “When did you take last physical examination? (excluding CHARLS physical examination)” in the questionnaire, if the respondent participated in a physical examination within the past year, the value was assigned as “1” to indicate active participation in a preventive health examination. If no physical examination was reported, a score of “0” was assigned, indicating no participation. Regular physical exercise was measured by the question: “Do you usually engage in physical activities for at least ten minutes weekly?” If a participant responded “no,” the value was assigned to “0,” indicating a lack of regular physical exercise. A response of “yes” was assigned a score of “1,” indicating regular engagement in physical exercise.

The five indicators mentioned above represent distinct behavioral domains that collectively form the construct of proactive health behavior, rather than being interchangeable reflections of a single underlying trait. Therefore, we conceptualized proactive health behavior as a formative construct. To avoid the fallacy of directly summing the five indicators, this study adopted the approach of the previous study [33]. It used the principal component factor method to obtain the factor loadings for each indicator. The following formula was then used to construct a proactive health behavior index based on the five factors:

  $$\begin{array}{c} \text{Proactive health behavior index}=\frac{\left(F1\times \text{factor }1+F2\times \text{factor }2+F3\times \text{factor }3+F4\times \text{factor }4+F5\times \text{factor }5\right)}{\left(\text{Sum of the }F\text{ coefficient}\right)}. \end{array}$$

A 0–1 normalization process is applied to generate the proactive health behavior index, a continuous variable with a value range of 0–10. The larger the value is, the higher the individual's proactive health behavior score.

#### 2.2.4. Control Variables

Several covariates identified in the literature as relevant to internet use and depressive symptoms were used as confounders. We included age (a continuous variable), gender (1 for male and 0 for female), marital status (1 for married or partnered and 0 for otherwise), and residence (0 for urban and 1 for rural). SES was assessed by the current work status (not currently working = 0, agricultural job = 1, and nonagricultural job = 2) [51], the highest educational level (categories including below elementary school = 0, elementary school = 1, middle school = 2, and high school or above = 3), and household income, divided into five quintiles. Besides, we included functional impairment as having difficulties in performing any instrumental activities of daily living (IADLs, which include managing money, taking medications, shopping for groceries, preparing hot meals, and household chores) and chronic disease status (1 for having at least one chronic disease and 0 for having no chronic disease). Consistent with existing studies and to reduce model complexity, all covariates were derived from the 2015 wave [20, 52] and all these baseline covariates were included in all models to adjust for their potential confounding effects overtime.

### 2.3. Statistical Analysis

Given that CHARLS provides only cross-sectional weights and no official longitudinal weights, and considering our primary goal was to examine the bidirectional associations between internet use and depressive symptoms rather than generate population-level estimates, we applied cross-sectional weights only in descriptive analyses (Table S2) and used unweighted models in the main analysis. We began with descriptive statistics and correlation coefficients between internet use, proactive health behavior, and depressive symptoms. Next, we examined the association between internet use and depressive symptoms by constructing cross-lagged models. The cross-lagged model can uncover causal interrelations and mutual influences among variables overtime [53]. The full information maximum likelihood (FIML) estimation method with robust standard errors accounted for missing data and nonnormality [54]. Model fit was examined based on the chi-square test (*χ*^2^), the root mean square error of approximation (RMSEA), the comparative fit index (CFI), the Tucker–Lewis index (TLI), and the standardized root mean square residual (SRMR). Given the large sample size for this study, the *χ*^2^ value was not considered a good indicator of model fit. RMSEA ≤ 0.06, CFI and TFI ≥0.95, and SRMR ≤ 0.06 are generally considered to indicate good model fit [55]. Third, following the existing literature [56, 57], a three-wave cross-lagged mediation model design was employed to assess the mediating role of proactive health behavior. We estimated the significance of the indirect effect (*a* × *b*) by applying the bootstrap method with 5000 resamples to generate a 95% bias-corrected confidence interval (CI). This approach is more recommended and widely adopted for mediation analysis as it offers higher statistical power [58]. The mediation effect was considered significant if the 95% bootstrap CI did not include zero. In this study, descriptive statistics and correlation analyses were performed using STATA 17 and cross-lagged analyses were performed using Mplus 8.3.

## 3. Results

### 3.1. Descriptive Statistics

Table 1 provides descriptive statistics for the participants at T1 (2015). The mean age of the participants was 58.32 years, of whom 47.15% were male, 90.28% were married or partnered, and 62.32% lived in rural areas. Among these participants, only 12.05% had completed high school or higher education; the second income quintile (lower-middle) had the highest proportion (27.57%), and agricultural work was the most prevalent (38.28%). The average score of the IADL for the interviewer was 14.43. 77.84% of the participants had chronic diseases. Overall, the internet use percentage of middle-aged and older adults increased from 7.17% (T1) to 41.37% (T3), and the mean of depressive symptoms scores of the middle-aged and older adults who participated in the study continuously grew from 7.62 (T1) to 8.79 (T3). However, the mean scores of proactive health behavior fluctuated slightly.

### 3.2. Correlative Statistics

Table 2 presents the correlation between internet use, proactive health behavior, and depressive symptoms at T1, T2, and T3. Results revealed a significant positive correlation between internet use and proactive health behavior. Pronounced negative correlations were observed between internet use and depressive symptoms, as well as proactive health behavior and depressive symptoms.

### 3.3. The Cross-Lagged Analysis

To explore the causal relationships between internet use and depressive symptoms, we constructed cross-lagged panel models. Following previous studies [59, 60] we constructed Model 1 without constraints. We subsequently constructed Model 2–Model 5, where autoregressive effects, lag effects, and correlation coefficients were set to be equal for the nonstandardized coefficients of the same path. Then, we applied the nested comparison approach to compare the fit for each of the five models and selected the most parsimonious model (Table 3). The results indicate no significant difference between the constrained and unconstrained models [61], suggesting that the constraints did not significantly weaken the model's goodness of fit. Therefore, the tests did not reject the stationarity assumption that the autoregressive and cross-lagged associations overtime were identical. Thus, we accepted the simplest model (Model 5) and reported only results from this model.

**Figure 2:**
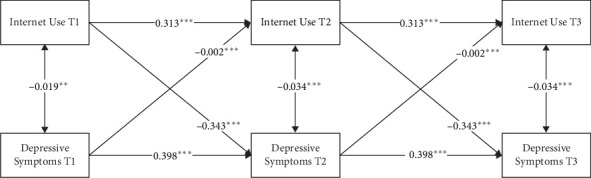
Path coefficients in Model 5. Single-headed arrows represent regression paths. Double-headed arrows represent correlations. All path coefficients were unstandardized and labeled as significant in the figure. *⁣*^*∗∗*^*p*  < 0.01, *⁣*^*∗∗∗*^*p*  < 0.001. This figure did not present covariates, residuals, and residual correlations for simplicity.

The results of this final model (Model 5) are presented in Figure 2. For the autoregressive effects, the initial internet use significantly predicted subsequent internet use (*b* = 0.313, *p* < 0.001); similarly, the initial depressive symptoms predicted subsequent depressive symptoms significantly (*b* = 0.398, *p* < 0.001). For the cross-lagged effects, internet use and depressive symptoms significantly affected each other. Specifically, internet use significantly predicted a decrease in depressive symptoms (*b* = −0.343, *p* < 0.001), and similarly, higher levels of depressive symptoms significantly predicted less internet use (*b* = −0.002, *p* < 0.001). However, the cross-lagged effect of internet use on depressive symptoms was stronger than the effect in the opposite direction.

### 3.4. The Cross-Lagged Mediation Analysis

We employed a longitudinal mediation model to investigate whether proactive health behavior mediated the longitudinal association between internet use and depressive symptoms. We also compared the models' fitness before and after setting constraints (autoregressive effects, lag effects, and coefficients of the same path were set to be equal, respectively). The results indicated that the constrained model (all the same path constraints) fits well (RMSEA = 0.027, CFI = 0.996, TLI = 0.972, and SRMR = 0.012), suggesting that the constraints did not significantly weaken the models' fitness [61].

**Figure 3:**
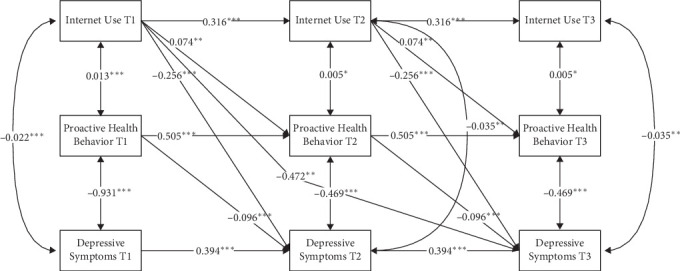
Path coefficients in the mediation model. Single-headed arrows represent regression paths. Double-headed arrows represent correlations. All path coefficients were unstandardized and labeled as significant in the figure. *⁣*^*∗*^*p*  < 0.05, *⁣*^*∗∗*^*p*  < 0.01, *⁣*^*∗∗∗*^*p*  < 0.001. For simplicity, covariates, residuals, and residual correlations were not presented in this figure.

Figure 3 displays the path coefficients and significance of internet use, proactive health behavior, and depressive symptoms. There was a significant negative correlation between internet use and depressive symptoms (*b* = −0.256, *p* < 0.001). Proactive health behavior also had a significant negative association with depressive symptoms (*b* = −0.096, *p* < 0.001). Internet use had a significant positive association with proactive health behavior (*b* = 0.074, *p* < 0.01). Besides, a significant negative correlation was observed between internet use at T1 and depressive symptoms at T3 (*b* = −0.472, *p* < 0.01). Notably, the indirect effect of proactive health behavior was statistically significant (*β* = 0.007, 95% CI: [−0.014, −0.001], *p* < 0.05), based on 5000 bias-corrected bootstrap resamples, suggesting that internet use was associated with reduced depressive symptoms partially through increased engagement in proactive health behavior (Table 4).

### 3.5. Heterogeneity Analysis

To investigate whether the cross-lagged effects and mediation effects differed between age groups (middle-aged adults: 45–59 years vs. older adults: ≥60 years) and subgroups by chronic condition (middle-aged adults and older adults with chronic diseases vs. middle-aged adults and older adults without chronic diseases), multigroup analysis (MGA) was conducted. The model comparison results indicated that there was no significant difference between different age groups (cross-lagged model: *χ*^2^ = 2.361, *p* = 0.307 > 0.05; cross-lagged mediation model: *χ*^2^ = 2.404, *p* = 0.493 > 0.05) and no significant difference between groups with and without chronic condition (cross-lagged model: *χ*^2^ = 3.725, *p* = 0.155 > 0.05; cross-lagged mediation model: *χ*^2^ = 2.527, *p* = 0.471 > 0.05). A detailed comparison of the path coefficients between subgroups by age and chronic condition is demonstrated in Tables S3 and S4, respectively. MGAs revealed that although some coefficients showed slight differences, no statistically significant differences were observed between age groups and those with and without chronic conditions.

### 3.6. Sensitivity Analysis

We performed sensitivity analyses to ensure the stability of our findings. First, we reestimated the CLPMs with digital exclusion as the independent variable (Tables S5 and S6 and Figures S1 and S2). Results show strong robustness and stability for the two measures of internet use. Second, to evaluate the potential impact of missing values on the results, we performed the analysis on the case samples (with multiple imputation), which yielded results consistent with the currently reported findings (Tables S7 and S8 and Figures S3 and S4). All of these sensitivity analyses collectively underscore the robustness of our findings.

## 4. Discussion

This study used a nationally representative longitudinal dataset to explore the bidirectional relationship between internet use and depressive symptoms among middle-aged and older adults. More importantly, this study ascertained one crucial underlying mechanism. Our findings demonstrated a significant bidirectional association between internet use and depressive symptoms among middle-aged and older adults overtime. Furthermore, our findings revealed that proactive health behavior partially mediated the relationship between internet use and depressive symptoms.

Our findings showed a bidirectional relationship between internet use and depressive symptoms among middle-aged and older adults. While internet use mitigated the inclination toward depressive symptoms, it was observed that middle-aged and older adults with higher levels of depressive symptoms were less likely to use the internet. Our findings, which are partly in line with previous cross-sectional studies [14, 62], confirmed that internet use was associated with a decrease in depressive symptoms among middle-aged and older adults. Besides, similar to a study on Finland's older migrants [63], our findings verified that middle-aged and older adults exhibiting a higher level of depressive symptoms were less likely to use the internet in China. Additionally, the findings also align with a recent study on older adults [20]. Regrettably, that study used only data from two waves of the CFPS (2016–2018), which limited the precise causal inference [20, 22]. Our study utilized the latest three waves of data from CHARLS, addressed the limitation that only two waves of data were insufficient in longitudinal designs and may lack credibility, expanded this finding from older adults to middle-aged and older adults, and further provided more powerful and precise evidence for the bivariate causal relationship between internet use and depressive symptoms. Higher levels of depressive symptoms were associated with less internet use, which may be attributed to the ability to use internet among individuals with high levels of depressive symptoms. Depression is a disorder characterized by a lowered mood and a reduction in energy, interest, and self-confidence [64]. The ability of older adults with depression to derive pleasure and focus is diminished, and they often experience significant fatigue even after the slightest exertion [65]. Coupled with age-related declines in cognition and physical functioning, middle-aged and older adults with depressive symptoms are more likely to experience more barriers when digital technology is used [51, 66].

Besides, our findings highlight the absence of internet access, and depressive symptoms could reciprocally exacerbate each other in a detrimental loop overtime; conversely, encouraging internet use and alleviating depressive symptoms may enhance the advantages progressively. Meanwhile, our studies found that internet use had a greater influence on change in depressive symptoms than vice versa. In the digital era, internet is increasingly permeating into middle-aged and older adults' lives, empowering their health behavior and services, enhancing their intrinsic capabilities, and playing a crucial role in developing and maintaining their functions for healthy aging. Western studies have indicated that internet use contributes to individuals acquiring more health-related knowledge and information, thereby improving their health status [67]. This further underscored the role of internet technology in empowering health. However, the current rate of internet use among middle-aged and older adults remains relatively low, undoubtedly inhibiting its effectiveness in promoting health empowerment. It is imperative to implement targeted strategies to bridge the digital divide and enhance the well-being of middle-aged and older adults. These include bolstering internet infrastructure, facilitating accessible connectivity, and promoting an age-friendly digital society. Engaging stakeholders such as the government, community organizations, and family units is crucial for fostering a cohesive and supportive digital ecosystem.

Our results also showed that internet use not only directly influences depressive symptoms among middle-aged and older adults but also indirectly improves their mental health by enhancing their proactive health behavior. Previous research has found that internet may enhance the health and well-being of older adults or middle-aged adults through various pathways, such as helping them maintain close contact with friends and family members [10], increasing their levels of social support and social contact [68] and social capital [25], promoting their social engagement [69], and increasing their social trust [70]. The finding of this study enriches the research on how internet use benefits the health of middle-aged and older adults by revealing the potential mechanism from the perspective of health promotion. Internet is a pivotal tool for fostering “information dissemination,” which is instrumental in reducing the cost of information spread and broadening users' channels of information [71]. Internet use facilitates access to health information for older adults to acquire more health-related knowledge and improve their health production skills [6, 72]. According to the knowledge attitude practice (KAP) model, the acquisition and comprehension of knowledge leads to more positive attitudes, which in turn lead to better practices and behaviors [73–75]. By disseminating relevant health-related information, internet aids older adults in recognizing the importance of healthy practices and enhancing their health awareness [76], catalyzing the “practice” of health behaviors among older adults. This finding further confirmed the critical point of Grossman's theory, which argues that health is an “investment commodity,” to maintain an individual's health requires the exercise of subjective initiative and continuous “investment in health,” which includes inputs such as the utilization of medical care, food, and behavior [77]. As an essential investment in health, proactive health behavior practiced by individuals can promote the accumulation of health capital [77], improving middle-aged and older adults' health outcomes. Our findings revealed that proactive health behavior is an important pathway through which digital technology empowers the health of middle-aged and older adults, thus, providing more effective paths for promoting healthy aging. Achieving healthy aging not only relies on external forces to maintain the dignity and quality of life of older adults but also requires encouraging older adults to actively maintain their health through internal forces, such as health behavior [78, 79].

Moreover, our heterogeneity analysis indicated that no statistically significant differences in the estimated pathways between middle-aged and older adults, or between individuals with and without chronic diseases. These results partly align with prior studies [80, 81], suggesting that the observed associations are robust across subgroups by age and health condition. One possible explanation is that both internet use and proactive health behavior are universally accessible resources that provide comparable opportunities for health promotion across different population groups. On the one hand, the internet facilitates access to services such as telemedicine, online health management platforms, and mental health support, which help address mental health issues for individuals across all groups [82, 83]. On the other hand, proactive health behavior such as regular exercise, health examination, and active self-care serves as a common behavioral pathway through which individuals maintain or improve their mental health, regardless of age or chronic disease status [32, 43]. In the context of China's aging society, both middle-aged and older adults, as well as individuals with and without chronic diseases, may similarly benefit from engaging in proactive health behavior. The robustness of our findings across population subgroups further underscores the importance of recognizing and leveraging proactive health behavior as a critical intrinsic health resource for advancing national strategies to address population aging [43]. On the one hand, society should create a favorable external support environment for middle-aged and older adults to engage in proactive health behavior, thereby strengthening the social foundation of such behavior. On the other hand, it is necessary to promote the internet as an essential vehicle for shaping health consciousness and healthy behavior among middle-aged and older adults.

Admittedly, this study also has several limitations. First, the CHARLS lacks detailed information on internet use, such as online time and specific internet functions. Therefore, this study cannot distinguish the pattern or function of internet use in later life. Internet users may vary in their use intensity, activities engaged in, and benefits gained from internet use [17]. Future studies may further explore the relationships between different use intensities, online activities, and depressive symptoms. Second, the CHARLS questionnaire failed to encompass all possible proactive health behavior measures. This study employed an exploratory method, focusing on healthy lifestyles and self-health management, to construct a proactive health behavior index. Some vital health lifestyle indicators, such as sedentary behavior and information on diet, et cetera, have not been included in this research. Expanded research on other health outcomes and behavior is warranted to yield a more comprehensive understanding of Chinese middle-aged and older adults' proactive health behavior and its association with individual health outcomes. Additionally, our primary analyses were conducted without applying weights, which may limit the generalizability of the findings to the broader Chinese middle-aged and older adult population. Future research could benefit from developing or applying appropriate longitudinal weighting techniques to improve the representativeness and robustness of findings. Finally, while this study focused on the mediating role of proactive health behavior, future research may also explore whether such behaviors moderate the impact of internet use on depressive symptoms, particularly across different usage patterns and individual characteristics.

Despite such limitations, our study sheds light on the direction of the association between internet use and depressive symptoms among older adults and reveals the mediating role of proactive health behavior in the path from internet use to depressive symptoms. Policymakers and practitioners should pay more attention to encouraging middle-aged and older adults to adopt the internet, adopt proactive health behavior, and provide more support for middle-aged and older adults who suffer from severe depressive symptoms to adopt digital technologies so that they may take advantage of the potential social and health benefits gained from using new technologies.

## Figures and Tables

**Table 1:** Descriptive statistics of participants who completed all three waves (*N* = 11,332).

Variables	Mean	Standard deviation	*N* (%)
Independent/dependent variable			
Internet use (T1)	—	—	813 (7.17)
Internet use (T2)	—	—	1489 (13.14)
Internet use (T3)	—	—	4688 (41.37)
Depressive symptoms (T1)	7.62	6.22	—
Depressive symptoms (T2)	8.33	6.42	—
Depressive symptoms (T3)	8.79	6.51	—
Mediator			
Proactive health behavior (T1)	7.50	1.56	—
Proactive health behavior (T2)	7.56	1.57	—
Proactive health behavior (T3)	7.51	1.56	—
Controls			
Age, (T1, 45–105)	58.32	8.69	—
Gender (T1)			
Male	—	—	5343 (47.15)
Female	—	—	5989 (52.85)
Educational attainment (T1)			
Below elementary school	—	—	4144 (36.57)
Elementary school	—	—	3362 (29.67)
Middle school	—	—	2461 (21.72)
High school and above	—	—	1365 (12.05)
Household income level (T1)			
1^st^ quintile	—	—	2448 (21.60)
2^nd^ quintile	—	—	3124 (27.57)
3^rd^ quintile	—	—	2137 (18.86)
4^th^ quintile	—	—	2399 (21.17)
5^th^ quintile	—	—	1224 (10.80)
** **Current work status (T1)			
Not currently working	—	—	3154 (27.83)
Agricultural job	—	—	4338 (38.28)
Nonagricultural job	—	—	3840 (33.89)
** **Marital status (T1)			
Otherwise	—	—	1102 (9.72)
Married or partnered	—	—	10,230 (90.28)
Residence (T1)			
Urban	—	—	4270 (37.68)
Rural	—	—	7062 (62.32)
IADL (T1, 0–15)	14.43	1.69	—
Chronic diseases (T1)			
YES	—	—	8821 (77.84)
No	—	—	2511 (22.16)

*Note*: T1 = Time 1, 2015; T2 = Time 2, 2018; T3 = Time 3, 2020.

**Table 2:** Correlation analysis for the independent/dependent/mediator variables.

Variables	1	2	3	4	5	6	7	8	9
1 IU (T1)	1	—	—	—	—	—	—	—	—
2 IU (T2)	0.452*⁣*^*∗∗∗*^	1	—	—	—	—	—	—	—
3 IU (T3)	0.307*⁣*^*∗∗∗*^	0.408*⁣*^*∗∗∗*^	1	—	—	—	—	—	—
4 PHB (T1)	0.045*⁣*^*∗∗∗*^	0.061*⁣*^*∗∗∗*^	0.035*⁣*^*∗∗*^	1	—	—	—	—	—
5 PHB (T2)	0.037*⁣*^*∗∗∗*^	0.033*⁣*^*∗∗∗*^	0.023*⁣*^*∗∗*^	0.603*⁣*^*∗∗∗*^	1	—	—	—	—
6 PHB (T3)	0.032*⁣*^*∗∗∗*^	0.037*⁣*^*∗∗∗*^	0.031*⁣*^*∗∗∗*^	0.587*⁣*^*∗∗∗*^	0.625*⁣*^*∗∗∗*^	1	—	—	—
7 DS (T1)	−0.107*⁣*^*∗∗∗*^	−0.126*⁣*^*∗∗∗*^	−0.134*⁣*^*∗∗∗*^	−0.074*⁣*^*∗∗∗*^	−0.076*⁣*^*∗∗∗*^	−0.067*⁣*^*∗∗∗*^	1	—	—
8 DS (T2)	−0.109*⁣*^*∗∗∗*^	−0.126*⁣*^*∗∗∗*^	−0.126*⁣*^*∗∗∗*^	−0.032*⁣*^*∗∗*^	−0.090*⁣*^*∗∗∗*^	−0.069*⁣*^*∗∗∗*^	0.532*⁣*^*∗∗∗*^	1	—
9 DS (T3)	−0.135*⁣*^*∗∗∗*^	−0.147*⁣*^*∗∗∗*^	−0.166*⁣*^*∗∗∗*^	−0.046*⁣*^*∗∗*^	−0.050*⁣*^*∗∗∗*^	−0.066*⁣*^*∗∗∗*^	0.530*⁣*^*∗∗∗*^	0.565*⁣*^*∗∗∗*^	1

*Note*: T1 = Time 1, 2015; T2 = Time 2, 2018; T3 = Time 3, 2020.

Abbreviations: DS, depressive symptoms; IU, internet use; PHB, proactive health behavior.

*⁣* ^*∗*^ *p* < 0.05.

*⁣* ^*∗∗*^ *p* < 0.01.

*⁣* ^*∗∗∗*^ *p* < 0.001.

**Table 3:** Model fit indices and nested model comparisons.

Models	Model fits					Model comparisons				
	*χ* ^2^ (df)	RMSEA	CFI	TLI	SRMR	Pairs	*∆*RMSEA	*∆*CFI	*∆*TLI	*∆*SRMR
Model 1	7.737 (2)	0.016	1.000	0.991	0.002	—	—	—	—	—
Model 2	7.672 (2)	0.016	1.000	0.991	0.002	2vs1	0.000	0.000	0.000	0.000
Model 3	8.285 (4)	0.010	1.000	0.997	0.002	3vs1	0.006	0.000	0.006	0.000
Model 4	7.573 (3)	0.012	1.000	0.995	0.002	4vs1	0.004	0.000	0.004	0.000
Model 5	8.081 (5)	0.007	1.000	0.998	0.002	5vs1	0.009	0.000	0.007	0.000

*Note*: Model 1, unconstrained baseline model; Model 2, model with all autoregressive paths fixed to be time-invariant; Model 3, model with all cross-lagged paths fixed to be time-invariant; Model 4, model with all T2–T3 correlated changes fixed to be time-invariant; Model 5, model with all autoregressive paths, cross-lagged paths, and T2–T3 correlated changes fixed to be time-invariant; *χ*^2^, chi-square test; *Δ*, change in parameter.

Abbreviations: CFI, comparative fit index; df, degrees of freedom; RMSEA, root mean square error of approximation; SRMR, standardized root mean square residual; TLI, Tucker–Lewis index.

**Table 4:** Mediation analyses for the mediation model.

Indirect paths	Effect	Boot SE	Boot 95% CI lower	Boot 95% CI upper
IU (T1)-IU (T2)-DS (T3)	−0.081*⁣*^*∗*^	0.036	−0.152	−0.010
IU (T1)-PHB (T2)-DS (T3)	−0.007*⁣*^*∗*^	0.003	−0.014	−0.001
IU (T1)-DS (T2)-DS (T3)	−0.101*⁣*^*∗*^	0.045	−0.188	−0.013

*Note*: T1 = Time 1, 2015; T2 = Time 2, 2018; T3 = Time 3, 2020. Indirect effects were unstandardized. 5000 bootstrap replicates were performed.

Abbreviations: Boot, bootstrap; DS, depressive symptoms; IU, internet use; PHB, proactive health behavior; SE, standard error.

*⁣* ^*∗*^ *p* < 0.05.

## Data Availability

This study uses open data. Researchers can apply for and download the data from the website of CHARLS (https://charls.charlsdata.com/pages/data/111/zh-cn.html).

## Supporting Information

Supporting Information Main analysis: Table S1. Fit indices for the confirmatory factor analysis of the 10-item CES-D scale. Table S2. Descriptive statistics of participants who completed all three waves (*N* = 11,167, weighted). Table S3. Results of multiple-group analysis between middle-aged adults and older adults. Table S4. Results of multiple-group analysis between middle-aged adults with and without chronic diseases. Sensitivity analysis 1: Table S5. Model fit indices and nested model comparisons. Figure S1. Path coefficients of the cross-lagged model. Figure S2. Path coefficients of the mediation model. Table S6. Mediation analyses for the mediation model. Sensitivity analysis 2: Table S7. Model fit indices and nested model comparisons. Figure S3. Path coefficients of the cross-lagged model. Figure S4. Path coefficients of the mediation model. Table S8. Mediation analyses for the mediation model.
