# Supplementary material for: Bidirectional Association Between Internet Use and Depressive Symptoms Among Middle-Aged and Older Adults in China: A Cross-Lagged Model of Proactive Health Behavior as the Mediating Role
Source: Depress Anxiety. 2025 Sep 22;2025:9391682. doi: 10.1155/da/9391682 (PMC12479151; doi:10.1155/da/9391682)
Supplement: Supporting Information — Main analysis: Table S1. Fit indices for the confirmatory factor analysis of the 10-item CES-D scale. Table S2. Descriptive statistics of participants who completed all three waves (N = 11,167, weighted). Table S3. Results of multiple-group analysis between middle-aged adults and older adults. Table S4. Results of multiple-group analysis between middle-aged adults with and without chronic diseases. Sensitivity analysis 1: Table S5. Model fit indices and nested model comparisons. Figure S1. Path coefficients of the cross-lagged model. Figure S2. Path coefficients of the mediation model. Table S6. Mediation analyses for the mediation model. Sensitivity analysis 2: Table S7. Model fit indices and nested model comparisons. Figure S3. Path coefficients of the cross-lagged model. Figure S4. Path coefficients of the mediation model. Table S8. Mediation analyses for the mediation model. [file 9391682.f1.docx]

**Supplement Material**

**Main analysis**

**Table S1.** Fit Indices for the Confirmatory Factor Analysis of the 10-item CES-D Scale

| In | χ²(df) | RMSEA | SRMR | CFI | TFI |
| --- | --- | --- | --- | --- | --- |
| T1 | 2234.10(33) | 0.061 | 0.033 | 0.951 | 0.934 |
| T2 | 2552.98(33) | 0.069 | 0.037 | 0.942 | 0.921 |
| T3 | 2556.75(33) | 0.069 | 0.036 | 0.944 | 0.924 |

*Note.*  T1= Time 1, 2015, T2= Time 2, 2018, T3= Time 3, 2020. χ²= Chi-Square Test, df = Degrees of Freedom, RMSEA= Root Mean Square Error of Approximation, SRMR= Standardized Root Mean Square Residual, CFI= Comparative Fit Index, TLI = Tucker-Lewis Index.

**Table S2.** Descriptive statistics of participants who completed all three waves. (N=11167, weighted)

| Variables | Mean | Standard deviation | N (%) |
| --- | --- | --- | --- |
| **Independent/dependent variable** |  |  |  |
| Internet use (T1) |  |  | 1099(9.85) |
| Internet use (T2) |  |  | 1885(16.89) |
| Internet use (T3) |  |  | 4943(44.27) |
| Depressive symptoms (T1) | 7.33 | 6.09 |  |
| Depressive symptoms (T2) | 8.05 | 6.30 |  |
| Depressive symptoms (T3) | 8.54 | 6.41 |  |
| **Mediator** |  |  |  |
| Proactive health behavior (T1) | 7.56 | 1.56 |  |
| Proactive health behavior (T2) | 7.59 | 1.56 |  |
| Proactive health behavior (T3) | 7.52 | 1.56 |  |
| **Controls** |  |  |  |
| **Age, (T1, 45-105)** | 58.51 | 8.78 |  |
| **Gender (T1)** |  |  |  |
| Male |  |  | 5308(47.53) |
| Female |  |  | 5859(52.47) |
| **Educational attainment (T1)** |  |  |  |
| Below elementary school |  |  | 3752(33.60) |
| Elementary school |  |  | 3244(29.05) |
| Middle school |  |  | 2546(22.79) |
| High school and above |  |  | 1625(14.56) |
| **Household income level (T1)** |  |  |  |
| 1st quintile |  |  | 223019.97) |
| 2nd quintile |  |  | 2761(24.73) |
| 3rd quintile |  |  | 2000(17.91) |
| 4th quintile |  |  | 2527(22.63) |
| 5th quintile |  |  | 1649(14.77) |
| **Current work status (T1)** |  |  |  |
| Not currently working |  |  | 3435(30.76) |
| Agricultural job |  |  | 3769(33.75) |
| Nonagricultural job |  |  | 3963(35.49) |
| **Marital status (T1)** |  |  |  |
| Otherwise |  |  | 1142(10.23) |
| Married or partnered |  |  | 10024(89.77) |
| **Residence (T1)** |  |  |  |
| Urban |  |  | 4270(37.68) |
| Rural |  |  | 7062(62.32) |
| **IADL (T1, 0-15)** | 14.47 | 1.62 |  |
| **Chronic diseases (T1)** |  |  |  |
| YES |  |  | 8688(77.80) |
| No |  |  | 2479(22.20) |

*Note.*  T1= Time 1, 2015, T2= Time 2, 2018, T3= Time 3, 2020. The weighted sample is slightly smaller due to missing weights for some respondents.

**Table S3.** Results of multiple-group analysis between middle-aged adults and older adults

| Paths | Middle-aged adults (n=6338) | Older adults (n=4994) | MD | *P* |
| --- | --- | --- | --- | --- |
|  | *β* | *β* |  |  |
| Cross-lagged paths |  |  |  |  |
| IU → DS | -0.288^**^ | -0.332^**^ | 0.044 | 0.865 |
| DS → IU | -0.002^***^ | -0.001^***^ | -0.001 | 0.128 |
| Mediation path |  |  |  |  |
| IU (T1)→PHB (T2)→DS (T3) | -0.005^*^ | -0.006^*^ | 0.001 | 0.942 |

*Note.* IU=Internet use, DS= Depressive symptoms, PHB= Proactive health behavior. T1= Time 1, 2015, T2= Time 2, 2018, T3= Time 3, 2020. ^***^*p* < 0.001, ^**^*p* < 0.01, ^*^*p* < 0.05. MD=mean difference of path coefficients.

**Table S4.** Results of multiple-group analysis between middle-aged adults with and without chronic diseases

| Paths | With chronic diseases (n=8821) | Without chronic diseases (n=2511) | MD | *P* |
| --- | --- | --- | --- | --- |
|  | *β* | *β* |  |  |
| Cross-lagged paths |  |  |  |  |
| IU → DS | -0.248^**^ | -0.578^**^ | 0.330 | 0.159 |
| DS → IU | -0.002^**^ | -0.003^***^ | 0.001 | 0.190 |
| Mediation path |  |  |  |  |
| IU (T1) →PHB (T2)→DS (T3) | -0.007^*^ | -0.006^*^ | 0.001 | 0.854 |

*Note.* IU=Internet use, DS= Depressive symptoms, PHB= Proactive health behavior. T1= Time 1, 2015, T2= Time 2, 2018, T3= Time 3, 2020. ^***^*p* < 0.001, ^**^*p* < 0.01, ^*^*p* < 0.05. MD=mean difference of path coefficient.

**Sensitivity Analysis 1**

We re-estimated the CLPMs with digital exclusion as the independent variable. The cross-lagged model fit indices are shown in Table S5, and the estimation results are shown in Figure S1. The cross-lagged mediation model also fit well (RMSEA = 0.029, CFI = 0.996, TLI = 0.972, SRMR = 0.012), and the estimation results are shown in Figure S2.

**Table S5.** Model fit indices and nested model comparisons.

| Models | Model Fits | | | | | | |  | Model comparisons | | | | | | |
| --- | --- | --- | --- | --- | --- | --- | --- | --- | --- | --- | --- | --- | --- | --- | --- |
|  | χ²(df) | RMSEA | | CFI | TLI | SRMR | | | | Pairs | ∆RMSEA | ∆CFI | ∆TLI | | ∆SRMR |
| Model 1 | 19.525(2) | | 0.028 | 0.999 | 0.977 | | 0.003 | | |  |  |  |  |  | |
| Model 2 | 19.113(2) | | 0.027 | 0.999 | 0.978 | | 0.003 | | | 2vs1 | 0.001 | 0.000 | 0.001 | 0.000 | |
| Model 3 | 23.092(4) | | 0.021 | 0.999 | 0.988 | | 0.004 | | | 3vs1 | 0.007 | 0.000 | 0.011 | 0.001 | |
| Model 4 | 28.163(3) | | 0.027 | 0.999 | 0.978 | | 0.004 | | | 4vs1 | 0.001 | 0.000 | 0.001 | 0.001 | |
| Model 5 | 30.928(5) | | 0.021 | 1.000 | 0.987 | | 0.004 | | | 5vs1 | 0.007 | 0.001 | 0.010 | 0.001 | |

*Note.* Model 1 = Unconstrained baseline model; Model2= Model with all autoregressive paths fixed to be time-invariant; Model 3= Model with all cross-lagged paths fixed to be time-invariant; Model 4= Model with all T2–T3 correlated changes fixed to be time-invariant; Model 5= Model with all autoregressive paths, cross-lagged paths and T2–T3 correlated changes fixed to be time-invariant; χ²= Chi-Square Test, df = Degrees of Freedom, RMSEA= Root Mean Square Error of Approximation, CFI= Comparative Fit Index, TLI = Tucker-Lewis Index, SRMR= Standardized Root Mean Square Residual, Δ = change in parameter.


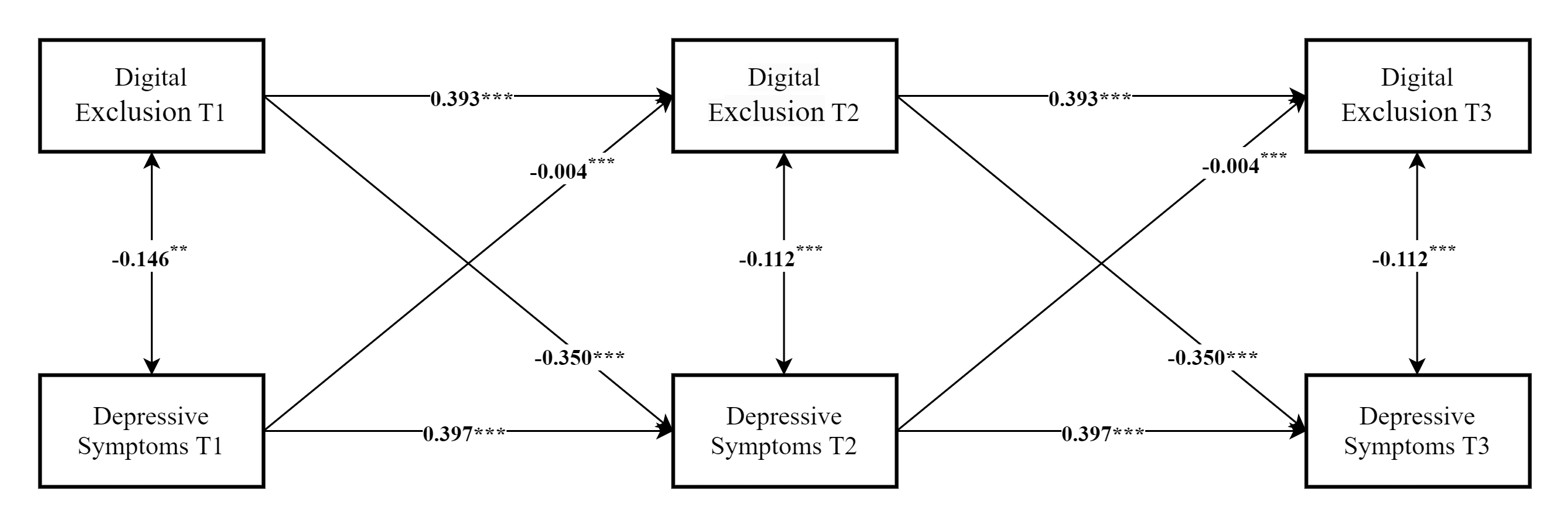


Figure S1. Path coefficients of the cross-lagged model

*Note.* Single-headed arrows represent regression paths. Double-headed arrows represent correlations. All path coefficients were unstandardized and labeled as significant in the figure. ^**^*p* < 0.01, ^***^*p* < 0.001. Figure S1 did not present covariates, residuals, and residual correlations for simplicity.


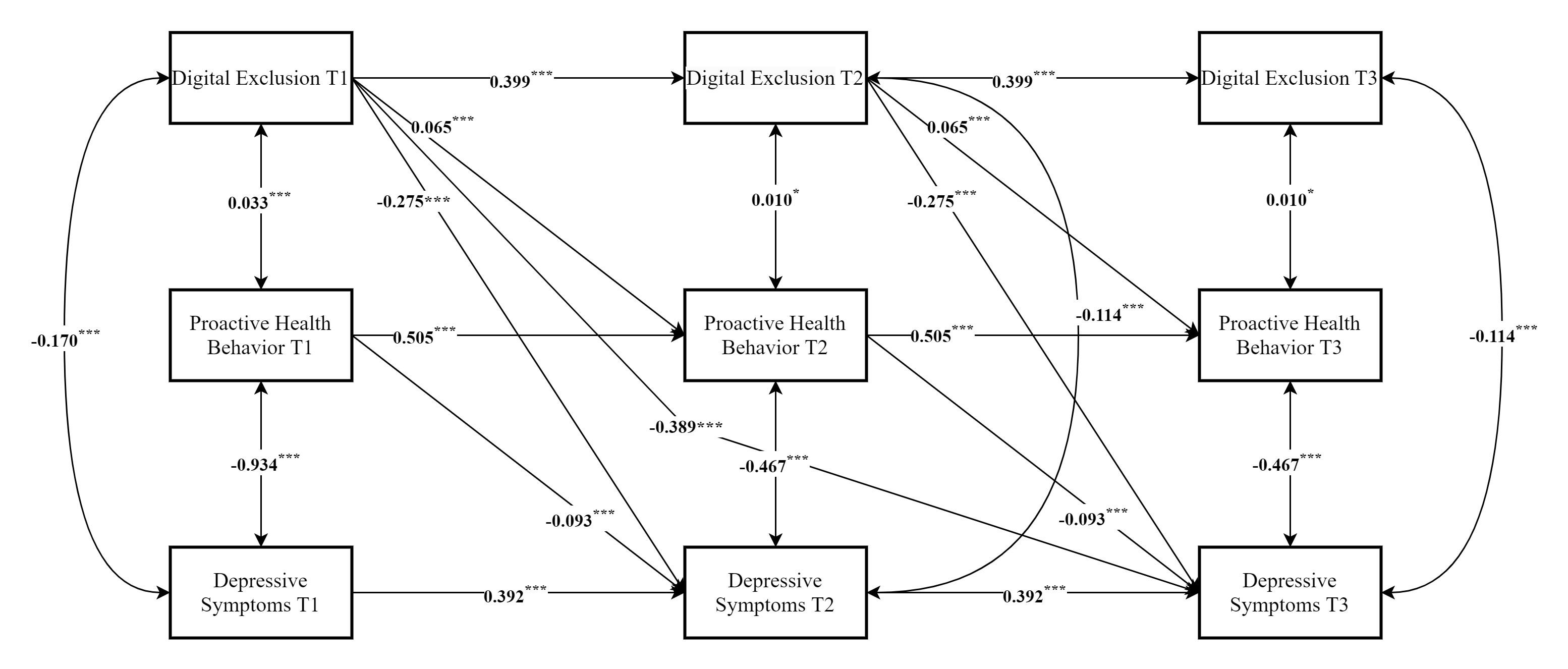
*Note.* Single-headed arrows represent regression paths. Double-headed arrows represent correlations. All path coefficients were unstandardized and labeled as significant in the figure. ^*^*p* < 0.05, ^**^*p* < 0.01, ^***^*p* < 0.001. For simplicity, covariates, residuals, and residual correlations were not presented in Figure S2.

Figure S2. Path coefficients of the mediation model

**Table S6.** Mediation analyses for the mediation model

| Indirect paths | Effect | Boot SE | Boot 95%CI Lower | Boot 95%CI Upper |
| --- | --- | --- | --- | --- |
| DE (T1)-DE (T2)- DS (T3) | -0.110^***^ | 0.025 | -0.159 | -0.062 |
| DE (T1)-PHB (T2)- DS (T3) | -0.006^**^ | 0.002 | -0.011 | -0.003 |
| DE (T1)-DS (T2)- DS (T3) | -0.108^***^ | 0.024 | -0.154 | -0.061 |

*Note.* DE= Digital exclusion, DS= Depressive symptoms, PHB= Proactive health behavior. T1= Time 1, 2015, T2= Time 2, 2018, T3= Time 3, 2020. Boot = Bootstrap. SE= Standard error. Indirect effects were unstandardized. 5000 bootstrap replicates were performed. ^*^*p* < 0.05, ^**^*p* < 0.01, ^***^*p* < 0.001.

**Sensitivity Analysis 2**

In order to examine the effects of missing data on the results, followed by previous studies(Di Gessa et al., 2016; Wang et al., 2023), We employed the multiple imputation (MI) techniques by Chained Equations techniques with 50 imputed datasets to estimate missing values for proactive health behaviors and other covariates (Murray, 2018). The results of analyses for each data set were then combined using Rubin’s rules (Little & Rubin, 2019).

The cross-lagged model fitness indices are shown in Table S7, and the estimation results are shown in Figure S3. The cross-lagged mediation model also fit well (RMSEA = 0.030, CFI = 0.995, TLI = 0.965, SRMR = 0.012), and the estimation results are shown in Figure S4.

**Table S7.** Model fit indices and nested model comparisons.

| Models | Model Fits | | | | | | |  | Model comparisons | | | | | | |
| --- | --- | --- | --- | --- | --- | --- | --- | --- | --- | --- | --- | --- | --- | --- | --- |
|  | χ²(df) | RMSEA | | CFI | TLI | SRMR | | | | Pairs | ∆RMSEA | ∆CFI | ∆TLI | | ∆SRMR |
| Model 1 | 10.149(2) | | 0.018 | 1.000 | 0.989 | | 0.002 | | |  |  |  |  |  | |
| Model 2 | 9.996(2) | | 0.018 | 1.000 | 0.989 | | 0.002 | | | 2vs1 | 0.000 | 0.000 | 0.000 | 0.000 | |
| Model 3 | 10.513(4) | | 0.011 | 1.000 | 0.995 | | 0.002 | | | 3vs1 | 0.007 | 0.000 | 0.006 | 0.000 | |
| Model 4 | 9.789(3) | | 0.013 | 1.000 | 0.994 | | 0.002 | | | 4vs1 | 0.005 | 0.000 | 0.005 | 0.000 | |
| Model 5 | 10.074(5) | | 0.009 | 1.000 | 0.997 | | 0.002 | | | 5vs1 | 0.009 | 0.000 | 0.008 | 0.000 | |

*Note.* Model 1 = Unconstrained baseline model; Model2= Model with all autoregressive paths fixed to be time-invariant; Model 3= Model with all cross-lagged paths fixed to be time-invariant; Model 4= Model with all T2–T3 correlated changes fixed to be time-invariant; Model 5= Model with all autoregressive paths, cross-lagged paths and T2–T3 correlated changes fixed to be time-invariant; χ²= Chi-Square Test, df = Degrees of Freedom, RMSEA= Root Mean Square Error of Approximation, CFI= Comparative Fit Index, TLI = Tucker-Lewis Index, SRMR= Standardized Root Mean Square Residual, Δ = change in parameter.


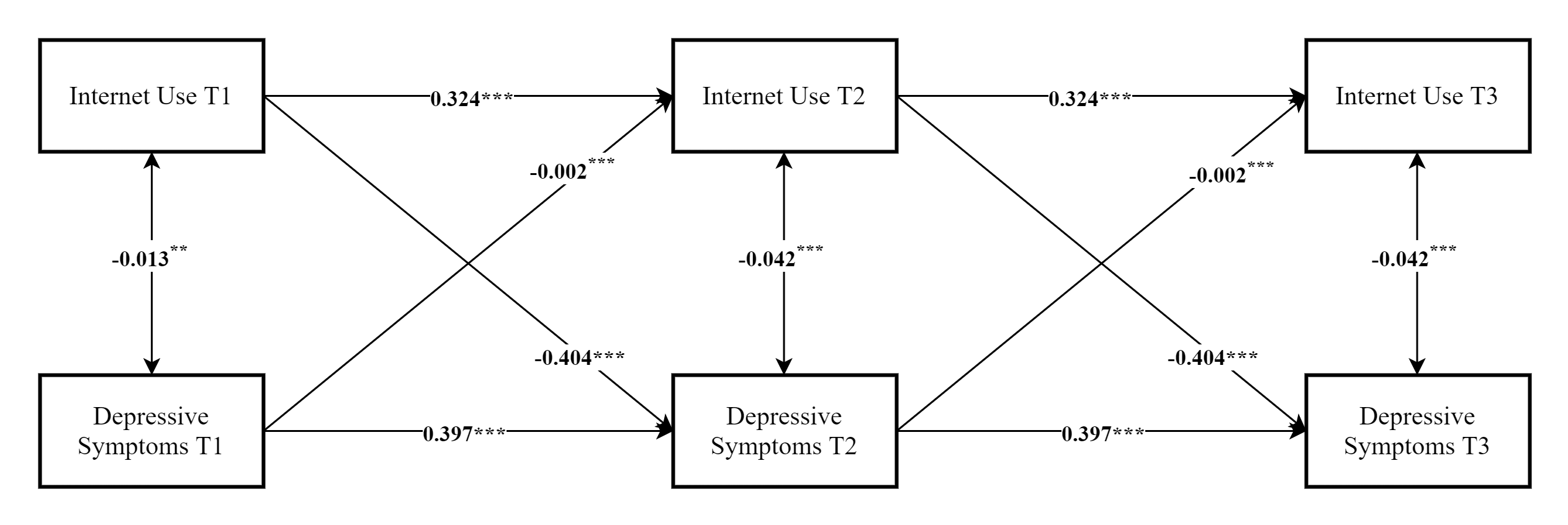


Figure S3. Path coefficients of the cross-lagged model

*Note.* Single-headed arrows represent regression paths. Double-headed arrows represent correlations. All path coefficients were unstandardized and labeled as significant in the figure. ^**^*p* < 0.01, ^***^*p* < 0.001. Figure S3 did not present covariates, residuals, and residual correlations for simplicity.


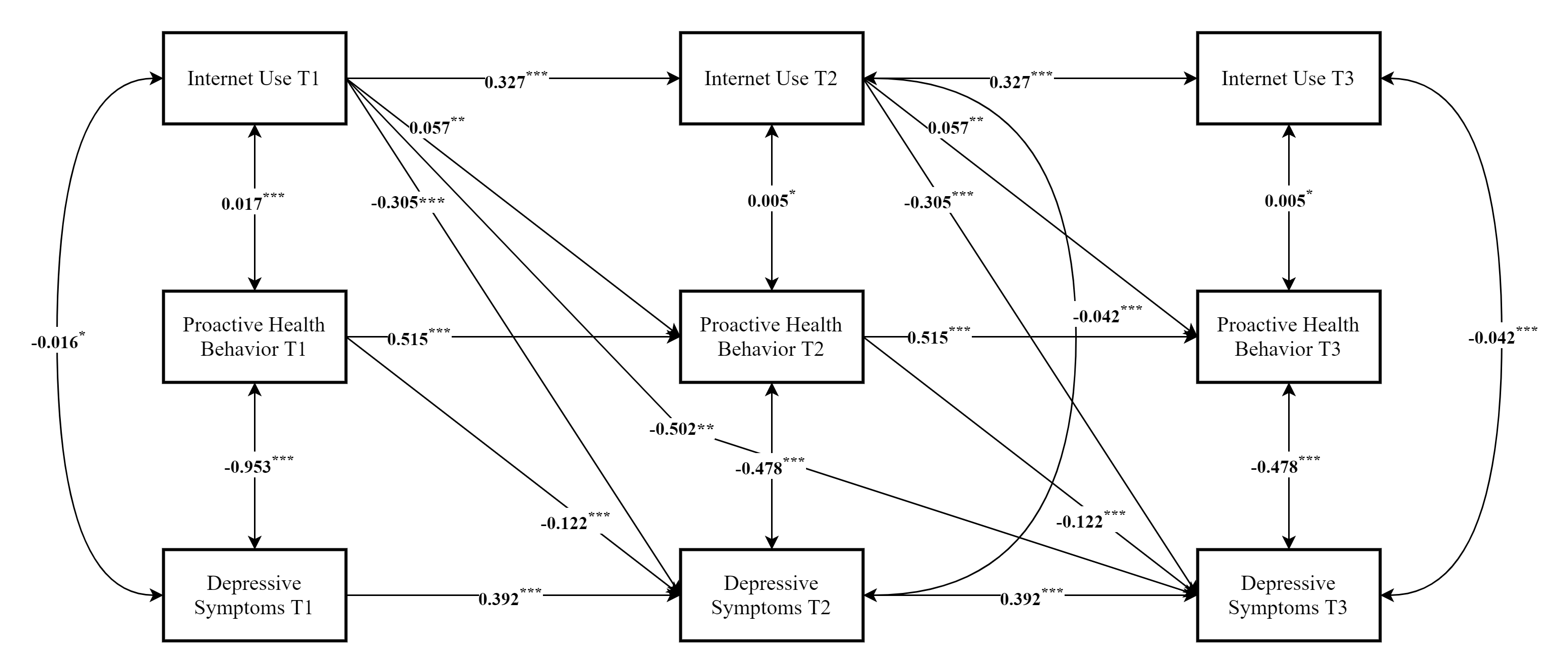


Figure S4. Path coefficients of the mediation model

*Note.* Single-headed arrows represent regression paths. Double-headed arrows represent correlations. All path coefficients were unstandardized and labeled as significant in the figure. ^*^*p* < 0.05, ^**^*p* < 0.01, ^***^*p* < 0.001. For simplicity, covariates, residuals, and residual correlations were not presented in Figure S4.

**Table S8.** Mediation analyses for the mediation model

| Indirect paths | Effect | Boot SE | Boot 95%CI Lower | Boot 95%CI Upper |
| --- | --- | --- | --- | --- |
| IU (T1)-IU (T2)- DS (T3) | -0.100^**^ | 0.037 | -0.172 | -0.028 |
| IU (T1)-PHB (T2)- DS (T3) | -0.007^*^ | 0.003 | -0.014 | -0.001 |
| IU (T1)-DS (T2)- DS (T3) | -0.120^**^ | 0.004 | -0.206 | -0.047 |

*Note.* IU= Internet use, DS=Depressive symptoms, PHB= Proactive health behavior. T1= Time 1, 2015, T2= Time 2, 2018, T3= Time 3, 2020. Boot = Bootstrap. SE= Standard error. Indirect effects were unstandardized. 5000 bootstrap replicates were performed. ^*^*p* < 0.05, ^**^*p* < 0.01, ^***^*p* < 0.001.

**Reference:**

Di Gessa, G., Glaser, K., & Tinker, A. (2016). The Health Impact of Intensive and Nonintensive Grandchild Care in Europe: New Evidence From SHARE. *The Journals of Gerontology Series B: Psychological Sciences and Social Sciences*, *71*(5), 867–879. https://doi.org/10.1093/geronb/gbv055

Little, R., & Rubin, D. (2019). Statistical Analysis with Missing Data, Third Edition. *Wiley Series in Probability and Statistics*. https://doi.org/10.1002/9781119482260

Murray, J. S. (2018). Multiple Imputation: A Review of Practical and Theoretical Findings. *Statistical Science*, *33*(2), 142–159. https://doi.org/10.1214/18-STS644

Wang, H., Liu, H., Wu, B., & Hai, L. (2023). The Association Between Trajectories of Perceived Unmet Needs for Home and Community-Based Services and Life Satisfaction Among Chinese Older Adults: The Moderating Effect of Psychological Resilience. *Research on Aging*, 01640275231203608. https://doi.org/10.1177/01640275231203608
